# Arousal Modulates Retinal Output

**DOI:** 10.1016/j.neuron.2020.04.026

**Published:** 2020-08-05

**Authors:** Sylvia Schröder, Nicholas A. Steinmetz, Michael Krumin, Marius Pachitariu, Matteo Rizzi, Leon Lagnado, Kenneth D. Harris, Matteo Carandini

**Affiliations:** 1 UCL Institute of Ophthalmology, University College London, London WC1E 6BT, UK; 2 UCL Queen Square Institute of Neurology, University College London, London WC1E 6BT, UK; 3 HHMI Janelia Research Campus, Ashburn, VA 20147, USA; 4 School of Life Sciences, University of Sussex, Brighton BN1 9QG, UK

**Keywords:** vision, retina, arousal, locomotion, superior colliculus

## Abstract

At various stages of the visual system, visual responses are modulated by arousal. Here, we find that in mice this modulation operates as early as in the first synapse from the retina and even in retinal axons. To measure retinal activity in the awake, intact brain, we imaged the synaptic boutons of retinal axons in the superior colliculus. Their activity depended not only on vision but also on running speed and pupil size, regardless of retinal illumination. Arousal typically reduced their visual responses and selectivity for direction and orientation. Recordings from retinal axons in the optic tract revealed that arousal modulates the firing of some retinal ganglion cells. Arousal had similar effects postsynaptically in colliculus neurons, independent of activity in the other main source of visual inputs to the colliculus, the primary visual cortex. These results indicate that arousal modulates activity at every stage of the mouse visual system.

## Highlights

• Imaging technique reveals the activity of retinal synapses in the awake, intact brain
• The activity of retinal synapses depends on the animal’s level of arousal
• Arousal mostly reduces visual responses and selectivity
• Arousal affects presynaptic and postsynaptic neurons in retina and colliculus

## Teaser

Schröder et al. show that the output of retinal ganglion cells depends not only on visual input but also on the animal’s level of arousal. The effect of arousal propagates to downstream neurons in the midbrain, independent of cortical input.

## Introduction

The activity of sensory brain regions is influenced by the level of arousal (Busse, 2018, McGinley et al., 2015, Schneider, 2020, Shimaoka et al., 2018). In the mouse visual system, this influence has been observed in primary visual cortex (V1; Niell and Stryker, 2010), lateral geniculate nucleus (Aydın et al., 2018, Erisken et al., 2014), and superior colliculus (SC; Ito et al., 2017, Savier et al., 2019). Arousal affects both spontaneous and visually driven activity, increasing firing rates in some neurons and decreasing them in others (Erisken et al., 2014, Ito et al., 2017, Niell and Stryker, 2010, Stringer et al., 2019, Vinck et al., 2015).

Arousal, however, may affect vision even earlier, in the output of the retina. Indeed, the retina receives inputs from the rest of the brain (e.g., Lörincz et al., 2008, Repérant et al., 2006, Repérant et al., 2007). Moreover, behavioral state might affect retinal synapses in the midbrain through presynaptic modulation (Miller, 1998). To test this hypothesis, we developed an optical approach to measure the activity of retinal synapses during behavior, in the intact brain.

## Results

**Figure 1:**
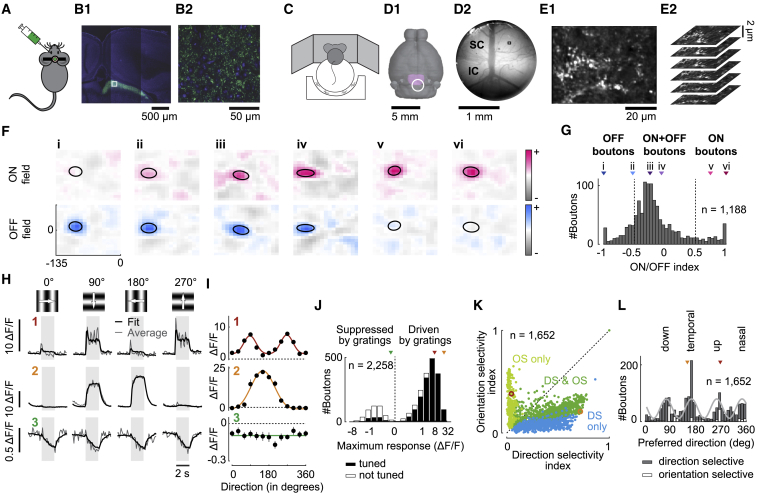
Visual Responses of Retinal Boutons Imaged in SC (A) SyGCaMP6f is injected into one eye. (B) Confocal images showing expression of SyGCaMP6f in synaptic boutons in the contralateral SC. (C) Mice were head-fixed on a treadmill surrounded by three monitors. (D) Positioning of the implant (circle) over superior colliculus (SC; purple), view through the implant showing SC and inferior colliculus (IC), and field of view of a typical two-photon imaging session of retinal boutons (rectangle). (E) Average frame of one two-photon imaging session (E1). Planes were imaged with 2-μm spacing to track boutons even during brain movements (E2). (F) Receptive fields of six boutons (i–vi), mapped with sparse sequences of white and black squares. Positions are in visual degrees relative to the front of the mouse and the height of the eyes. Ellipses outline the receptive fields at half height. (G) Distribution of ON/OFF indices across all boutons. For ease of description, we defined boutons as “ON” if their ON/OFF index was >0.5, “OFF” if it was <−0.5, and “ON+OFF” if it was intermediate. Triangles show ON/OFF indices of examples in (F). (H) Average (gray) and fitted (black) calcium responses of three boutons (1–3) in response to four sinusoidal gratings drifting in four directions. Gray shades show the times of stimulus presentation. (I) Direction tuning (mean ± SEM) and fitted tuning curves (solid lines) of the three boutons in (H) in trials where the pupil was small. (J) Distribution of maximum amplitudes in response to gratings across boutons. Boutons that are suppressed by gratings have negative maxima. Black bars represent boutons that are tuned to direction; white bars represent boutons that are not tuned. Triangles show maximum responses of examples in (H) and (I). (K) Orientation and direction selectivity indices of boutons that are selective only for orientation (light green), only for direction (blue), or for both orientation and direction (dark green). Circles show examples 1 and 2 in (H) and (I). (L) Distribution of preferred directions of boutons that are direction selective (gray bars) or only orientation selective (white bars). Fourth harmonic (light gray line) was fit using Fourier decomposition. Triangles show examples 1 and 2 in (H) and (I).

To measure retinal activity in the intact, awake brain, we imaged the synaptic boutons of retinal axons in the SC (Figures 1A–1F). We targeted the expression of a calcium indicator (SyGCaMP6f) to the axonal boutons of retinal ganglion cells (Dreosti et al., 2009, Liang et al., 2018) in contralateral superficial SC (Figures 1A and 1B). We placed mice on a treadmill (Figure 1C), and we performed two-photon imaging through an implant that accessed the posterior region of superficial SC without damaging the brain (Figures 1D, 1E, S1A, and S1B). We could thus measure the receptive fields of individual retinal boutons by imaging the intact brain (Figure 1F).

The visual responses of the retinal boutons resembled those of retinal ganglion cells (Figures 1G–1J). Consistent with findings in retina (Ratliff et al., 2010), OFF receptive fields were more common (mean ON/OFF index, −0.16; defined as relative difference between peaks of ON and OFF fields; Figure 1G and S1D). Gratings drove 80% of boutons (Figures 1H and 1I, boutons 1 and 2), most of which (91%) were tuned to orientation or direction (Figure 1J). Among tuned boutons, those highly selective for orientation were not selective for direction, and vice versa (p < 0.0001, permutation test; Figure 1K). This result differs from visual cortex, where selectivity for orientation and direction go hand in hand (Hubel and Wiesel, 1962, Lee et al., 2012, Niell and Stryker, 2008) but conforms to retina, where cells selective for direction have broad tuning for orientation (Grzywacz and Amthor, 2007). As in retina (Borst and Euler, 2011, Chen et al., 2014), direction selectivity was stronger in “ON+OFF” boutons than in “ON” (p = 1.83e-5) or “OFF” boutons (p = 0.001, linear mixed-effects model; Figures S1E and S1F), and the preferred directions clustered around the cardinal axes (up, down, nasal, and temporal) (Figures 1L and S1C). Other boutons (20%) were suppressed by gratings and unselective for direction or orientation (e.g., Figure 1H, bouton 3), echoing the properties of known retinal ganglion cell types (Jacoby and Schwartz, 2018). This suppression may involve the non-classical surround seen in retina (Solomon et al., 2006); 65% of boutons suppressed by gratings were driven by the smaller squares used to map receptive fields (as were 99% of the boutons driven by gratings). Only 26% of boutons suppressed by gratings were tuned to direction or orientation (Figure 1J).

**Figure 2:**
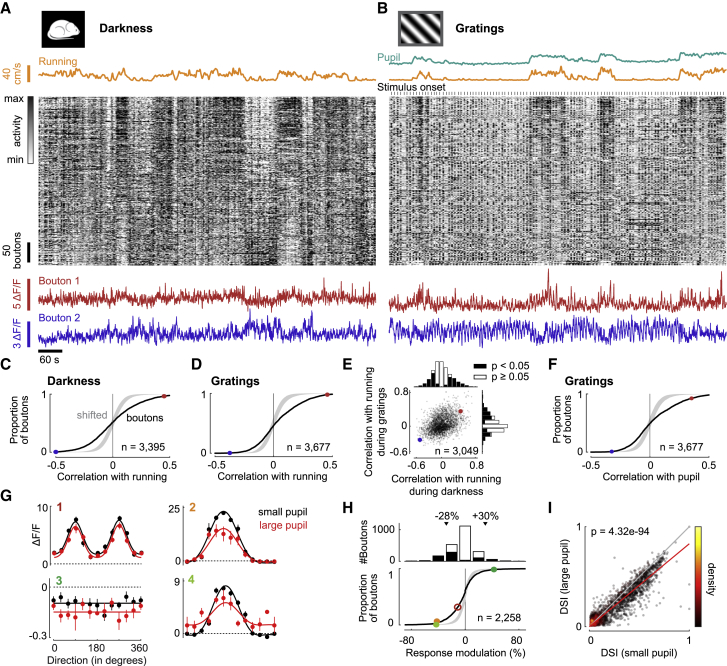
Activity in Retinal Boutons Varies with Arousal (A) Data from one experimental session recorded simultaneously during complete darkness showing running speed (yellow), calcium traces of retinal boutons (gray scale), and traces of two boutons (red and blue) that have positive and negative correlation with running. Calcium traces in gray scale were each *Z* scored and then sorted by mean correlation with running speed during darkness and during presentation of gratings (in B). (B) Data recorded from same retinal boutons as in (A) during presentation of gratings. In addition to (A), pupil size (green) and stimulus onsets (tick marks) are shown. (C) Correlation of retinal bouton calcium traces with running speed in darkness. Gray shade represents 2.5th to 97.5th percentile interval for null distribution generated by randomly shifting running trace against calcium traces. Dots show correlation strengths of example neurons in (A) and (B). (D) As in (C), but during presentation of gratings. (E) Correlation with running speed in darkness versus during visual stimulation for each bouton. Histograms show marginal distributions (same data as in C and D) for boutons with significant (black bars) and nonsignificant (white bars) correlations. (F) Correlation with pupil size during presentation of gratings. (G) Direction tuning (mean ± SEM) of four boutons during small (black) or large (red) pupil. Solid lines represent fitted tuning curves. Dotted lines represent 0 ΔF/F. Examples 1–3 are the same as in Figures 1H and 1I. (H) Distribution of arousal modulation for responses to gratings during small versus large pupil. Gray shade shows the 2.5th to 97.5th percentile interval of null distribution. Dots show modulations of examples in G (filled dots have significant modulations). Triangles (top) show mean values for boutons with significant positive or negative response modulations. (I) Direction selectivity index (DSI) during small versus large pupil for each bouton. Red line represents linear regression (linear mixed-effects model without intercept). Color of dots represents density in scatterplot.

Remarkably, the activity of retinal boutons also depended on arousal (Figures 2A–2F). We measured bouton activity in darkness and related it to the animal’s running speed, a common measure of arousal (McGinley et al., 2015). Running increased activity in many boutons and decreased it in many others (Figure 2A). Correlation with running speed was significant in 45% of the boutons (p < 0.05, shift test), positive in 23% of boutons, and negative in 22% of boutons (Figure 2C). We found similar correlation strengths with running speed while presenting drifting gratings (Figure 2B); 41% of retinal boutons showed significant correlations with running speed (p < 0.05, shift test), positive in 25% of the boutons and negative in the remaining 16% (Figure 2D). Results were consistent in darkness and during visual stimulation (r = 0.36, p = 7.19e-95, Student’s t test, n = 3,049 boutons; Figure 2E). When the pupil is not fully dilated (as is the case in darkness), it provides a measure of arousal (McGinley et al., 2015) that correlates with running speed (Figure S2A). Retinal boutons showed similar correlations with running speed and with pupil size (Figure S2B); 41% of retinal boutons showed significant correlations with pupil size (p < 0.05, shift test), positive in 23% of the boutons and negative in the remaining 18% (Figure 2F).

These effects of arousal on retinal boutons were not due to the increase in retinal illumination that accompanies pupil dilation. Such an increase would drive “ON” ganglion cells and suppress “OFF” ganglion cells, whereas we often observed the opposite: correlations with pupil were significantly negative in 20% of “ON” boutons and positive in 16% of “OFF” boutons (Figures 1F and S2Ki). Moreover, as we have seen, arousal had the same effects in darkness (Figure 2E), ruling out a role of retinal illumination.

Arousal typically reduced the boutons’ visual responses (Figures 2G and 2H). We separated trials of stimulus presentation into two groups (small pupil versus large pupil) based on the average pupil size during the trial. Arousal did not change the boutons’ preferred orientation (p > 0.05, circular paired t test) or direction (in 85% of boutons, the change in preferred direction was <20°; Figures 2G, S2E, and S2F). However, it did scale their visual responses additively and multiplicatively (Figure 2G). A model that allowed for both of these effects explained on average 47% (cross-validated) of response variance in tuned boutons. Allowing changes in preferred orientation or direction or in tuning width did not improve the cross-validated fits (p = 0.95, ANOVA). Arousal typically decreased the boutons’ peak visual response, quantified as the relative difference between responses during high and low arousal (responses were measured at the preferred direction for tuned boutons and averaged across stimuli for untuned boutons). Responses decreased with arousal in 26% of boutons, by 28% on average (p < 0.05, permutation test, Figure 2H). In contrast, only 5% of boutons significantly increased their visual response with arousal (by 30% on average). Changes in response amplitude were independent of preferred direction (p = 0.2811, ANOVA; Figure S2G). Boutons that were most affected at the peak visual response exhibited stronger correlations with pupil size (Figure S2H). Arousal on average decreased responses in boutons driven by gratings and increased responses in boutons suppressed by gratings (Figure S2Kii: p = 0.004, linear mixed-effects model; Figure S2Lii: p = 0.024, linear mixed-effects model). Also, arousal decreased responses more in boutons selective for both orientation and direction than in boutons selective for either orientation or direction (Figure S2Kiii: p = 0.00011, ANOVA; Figure S2Liii: p = 2.77e-5, ANOVA).

Arousal made retinal boutons less selective for direction and orientation (Figure 2I). The reduction in responses to optimal visual stimuli was not always matched by a reduction for other stimuli. In some boutons, in fact, arousal increased the average response across stimuli (e.g., bouton 4 in Figure 2G). Consequently, arousal generally reduced the tuning depth (decrease in 28% of boutons and increase in 3% of boutons), i.e., the difference between responses to preferred and null direction (p < 0.05, permutation test; Figure S2I). Moreover, arousal significantly decreased the boutons’ selectivity, reducing their direction selectivity index (DSI) by 17% (p = 4.32e-94, n = 2,256 boutons, 17 sessions, 8 mice, linear mixed-effects model without intercept; Figure 2I) and orientation selectivity index (OSI) by 11% (p = 5.28e-87, Figure S2D).

**Figure 3:**
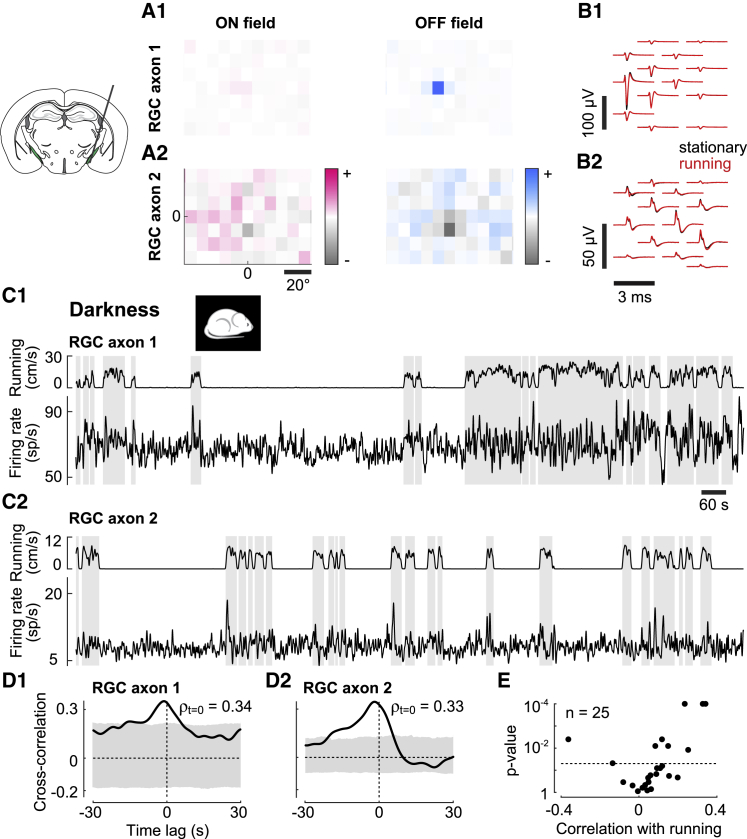
Effect of Arousal Is Present in Firing Rates of Retinal Ganglion Cells (A) ON and OFF receptive fields of example retinal axons 1 (A1) and 2 (A2). (B) Spike waveforms of axon 1 (B1) and axon 2 (B2) on multiple neighboring channels of probe when the animal was running (red) or stationary (black) (recorded in darkness). (C) Traces of running speed (top) and firing rate (bottom) of axon 1 (C1) and axon 2 (C2) recorded in darkness. Grey shades represent periods of running (≥1 cm/s). (D) Cross-correlograms between firing rate and running speed for axon 1 (D1) and axon 2 (D2). A positive lag denotes that firing rate is lagging running speed. Grey shade shows the 2.5th to 97.5th percentile interval of time-shifted data. (E) Correlation strengths (measured at lag zero) with running speed in darkness versus the p value of correlation.

To test whether these effects of arousal may originate in retina, we recorded the activity of retinal axons in the optic tract using Neuropixels probes (Jun et al., 2017), validating these recordings according to stringent criteria (Figures 3A and 3B). The optic tract is only ∼200 μm in diameter and lies ∼4 mm deep, so it is easy to miss. Even when the probe is well placed, it can only cover the optic tract with ∼20 recording sites. Furthermore, axons are tiny, so their signals are easily lost if they move relative to the probe, as is common during running. We thus adopted a spike sorting algorithm (Kilosort2) that can track spikes in the face of brain movements, and we curated its output and selected neurons based on stringent criteria for the quality of the recordings. The first criterion was anatomical: units were selected if histological reconstructions (Shamash et al., 2018) placed them in the region of the optic tract (Figures S3A and S3C). This criterion yielded 1,280 putative optic tract units. The second criterion was visual; units were selected if they responded reliably to rapidly flickering stimuli (Figures S3B and S3D) or had a clear spatial receptive field (Figure 3A) and short latency in response to grating stimuli. This criterion narrowed this sample to 49 units. The third criterion was electrophysiological; units were selected based on spike amplitude and lack of correlation between this amplitude and firing rate. This criterion narrowed the sample to 25 hard-won, stable, and visual units. To avoid biases, this rigorous selection was further validated by a second operator who was masked to the variables of interest, such as running speed. We then considered these variables and found, reassuringly, that running caused no changes in spike shape in any of the 25 retinal axons (Figure 3B).

These recordings revealed that arousal can significantly modulate the firing of retinal ganglion cells (Figures 3C–3E). Consider the firing rates of two retinal axons measured in complete darkness to avoid any confound due to pupil dilation (Figure 3C). In both axons, activity varied with locomotion. Indeed, for both axons, the cross-correlation between firing rate and running speed shows a significant positive peak around lag zero (Figure 3D). To test whether these correlations were larger than expected by chance, we measured correlations with surrogate running speed traces that were randomly shifted in time (Figure 3D, gray bands). Correlations were significant in 9 of the 25 axons (significantly positive in 7 axons and significantly negative in 2 of 25 axons) (p < 0.05, shift test; Figure 3E). This result would be unlikely if due to chance (p = 7.6e-10, Fisher’s combined probability test) and did not seem to be particular to axons with given receptive field properties (Figure S3E).

**Figure 4:**
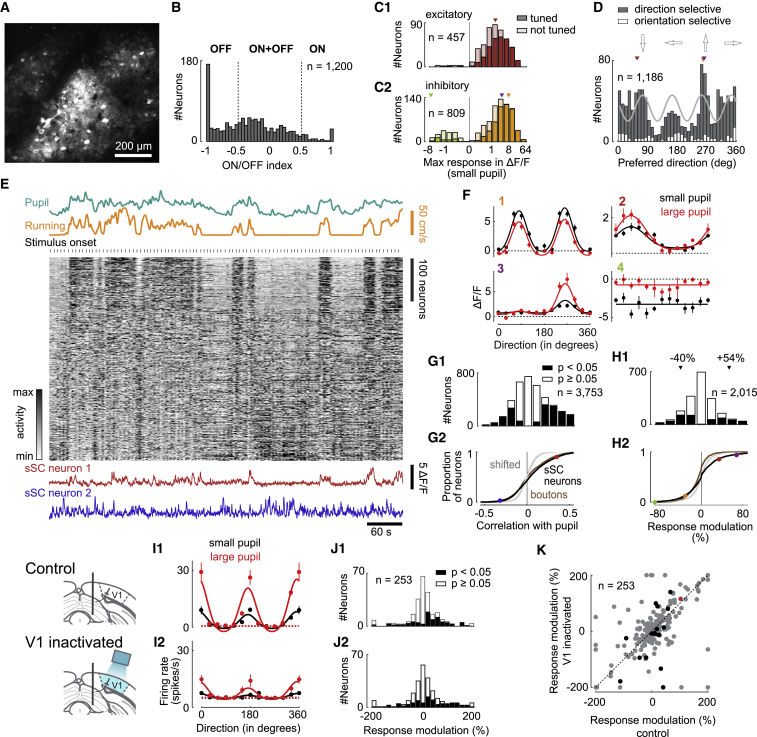
Visual Responses and Effect of Arousal on Neurons in SC (A) Average frame of two-photon imaging data shown in (H). (B) Distribution of ON/OFF indices across SC neurons. (C) Distribution of maximum amplitudes in response to gratings for excitatory (C1) and inhibitory neurons (C2). Dark bars represent neurons tuned to direction; light bars represent untuned neurons. Triangles show responses of examples in F. (D) Distribution of preferred directions of neurons that are direction selective (gray bars) or only orientation selective (white bars). Fourth harmonic (light gray line) was fit using Fourier decomposition. Triangles show preferred directions of examples 1–3 in (F). (E) Data from one experimental session recorded during presentation of gratings. Calcium traces in gray scale were each *Z* scored and then sorted by correlation with pupil size in the first half of data; only the second half is presented to show the robustness of correlations. (F) Direction tuning (mean ± SEM) of four neurons during trials where the pupil was small (black) or large (red). (G) Correlations of SC neurons with pupil size during presentation of gratings. Shaded area (G2): 2.5-97.5 percentile interval of null distribution. Brown line shows correlations of retinal boutons (same as in Figure 2F). Dots show correlations of example neurons in E. (H) Distribution of response modulation (measured and null). Dots: values of examples in F. (I) Direction tuning (mean ± SEM) of SC neurons during trials where the pupil was small (black) or large (red). Tuning curves were measured during control conditions (I1) and V1 inactivation (I2). Dotted lines show baseline firing rates. (J) Distribution of response modulations during control conditions (J1) and V1 inactivation (J2). (K) Response modulation during control conditions versus V1 inactivation for neurons with nonsignificant changes in response modulation (p < 0.05, permutation test, gray dots) and neurons with significant changes (black dots). Red dot marks values of neuron in (I).

We next asked if similar effects of arousal extend postsynaptically to the visual activity of SC neurons (Figures 4A–4D). We expressed GCaMP6f in SC neurons in mice expressing red TdTomato in inhibitory neurons and recorded neuronal activity with two-photon imaging (Figure 4A). Similar to retinal boutons, SC neurons tended to have stronger OFF subfields (Figures 4B, S4A, S4B, S4D, and S4G). Most SC neurons (88%) were driven by gratings (Figure 4C), and among these, many were tuned to stimulus direction (70%; Figures S4C, S4E, S4F, S4H, and S4I). As in retinal boutons, the preferences of these neurons clustered around cardinal directions (Figures 4D and S4J). The remaining neurons were suppressed by gratings, and among these, most (69%) were not tuned to stimulus direction (Figure 4C). As in retinal boutons, suppression by gratings was likely enhanced by surround suppression, because many of the neurons that were suppressed by gratings were driven by small squares (41% versus 97% among the neurons driven by gratings). Remarkably, almost all neurons (91%) that were suppressed by gratings were inhibitory (Figure 4C).

Arousal caused similar effects in SC neurons and retinal synapses (Figures 4E–4H). Across 3,753 SC neurons, 43% had significant correlations (28% positive and 15% negative) with pupil size (p < 0.05, shift test; Figure 4G). The distribution of correlations with pupils size closely matched that of retinal boutons (Figure 4G2). Correlations with pupil size were largely independent of visual drive (Figure S4L), and they resembled correlations with running speed (Figure S4M). As in retinal boutons, arousal decreased direction and orientation selectivity in SC neurons by 10% (p = 1.50e-275 for DS and p = 1.64e-118 for OS; n = 2,015 neurons, 10 sessions, six mice, linear mixed-effects model without intercept; Figures S4N and S4O), without changing preferred directions and orientations (p > 0.05, circular paired t test; Figures S4P and S4Q). Arousal also significantly affected peak visual responses, increasing them in 17% of SC neurons (p < 0.05, permutation test, average increase of 54%) and decreasing them in 15% of SC neurons (average decrease of 40%; Figures 4H and S4R). The increase in visual responses with arousal was significantly larger in excitatory than inhibitory SC neurons (Figures S4Tii, S4Uii, and S4Vii). Similar to retinal boutons, arousal decreased visual responses more in neurons selective to both orientation and direction than in neurons selective to either orientation or direction (Figures S4Tiii and S4Uiii).

These effects of arousal persisted when we inactivated the other main source of visual inputs to SC, the V1 (Figures 4I–4K). V1 projects to SC (e.g., Wang and Burkhalter, 2013) and influences its visual responses (e.g., Zhao et al., 2014). Given that arousal modulates V1 responses, this modulation might be passed on from V1 to SC. To test this hypothesis, we silenced optogenetically a large part of V1 (Figures S4W and S4X) in mice that expressed ChR2 in cortical parvalbumin-expressing inhibitory neurons (Cardin et al., 2009, Lien and Scanziani, 2013). Simultaneously, we recorded SC activity with Neuropixels probes. Inactivating V1 had diverse effects on visual responses of SC neurons, decreasing them on average (Zhao et al., 2014) independent of the level of arousal (p < 0.05, Wilcoxon signed rank test; Figures S4Y and S4Z). V1 inactivation did not, however, diminish the effects of arousal on SC neurons. For example, V1 inactivation reduced the responses of the neuron in Figure 4I but did not change the relative effect of arousal on the responses. During V1 inactivation, arousal still modulated visual responses of 38% of SC neurons (p < 0.05, permutation test; Figure 4J2), the same fraction as in control conditions (Figures 4I1 and 4J1). Only 2% of SC neurons were affected less by arousal during V1 inactivation than in control conditions (p < 0.05, permutation test; Figure 4K). The effects of arousal in SC, therefore, are consistent with the effects seen in retinal boutons and do not appear to be mediated by top-down influences from V1.

## Discussion

Our results reveal that the activity of retinal synaptic boutons and even retinal ganglion cells depends on the level of arousal.

These effects could be mediated by the projections that the brain sends to the retina. Such retinopetal projections are present in birds (Wilson and Lindstrom, 2011), fish (Esposti et al., 2013), and other vertebrates (Repérant et al., 2007, Repérant et al., 2006). In rodents, they include histaminergic fibers from hypothalamus and serotonergic fibers from the dorsal raphe (Gastinger et al., 2006), which signal behavior and arousal (Jacobs and Azmitia, 1992, Ranade and Mainen, 2009, Vanni-Mercier et al., 2003). Stimulation of the dorsal raphe enhances global visual responses in the retina (Lörincz et al., 2008), whereas blockade of the optic nerve can have opposite effects (Molotchnikoff et al., 1989).

The effects of arousal on retinal ganglion cells may also result from additional factors, such as changes in arterial oxygen tension, glucose concentration, and intraocular pressure (Ames et al., 1992, Yancey and Linsenmeier, 1988). Such mechanisms, however, may be too slow to fully explain the effects that we measured. Similarly, some of the effects of arousal may be due to the changes in light level that accompany pupil dilation, but these changes could not explain the results we obtained in darkness.

Additional mechanisms could operate at retinal boutons in superior colliculus, such as through presynaptic modulation (Chen and Regehr, 2003, Miller, 1998) by neuromodulators such as serotonin (Beitz et al., 1986, Mooney et al., 1994). Presynaptic modulation could also explain the effects of arousal on retinal boutons in the geniculate nucleus recently measured by Liang et al. (2020). However, it could not explain the changes in firing rate that we observed in the optic tract.

The effects we observed in retinal boutons, therefore, may be due to a combination of local and distal mechanisms. To disentangle this combination, future studies face a substantial challenge: to manipulate retinopetal or collicular neuromodulation without interfering with dopaminergic and serotonergic neurons that are located inside the retina (Dhande et al., 2015, Masson, 2019).

The effects of arousal in retinal axons and synapses are likely to contribute to the effects of arousal in SC neurons. We found that arousal affected similar proportions of retinal boutons and SC neurons, enhancing or suppressing spontaneous activity of both populations in similar proportions. Also, arousal mainly decreased direction and orientation selectivity, both in retinal boutons and SC neurons. However, while most retinal boutons decreased visual responses with arousal, only half of the SC neurons decreased their responses, with the other half increasing their responses. This heterogeneity agrees with previous findings in the SC (Ito et al., 2017, Savier et al., 2019) and with effects of arousal in other visual areas (Erisken et al., 2014, Ito et al., 2017, Stringer et al., 2019, Vinck et al., 2015). Perhaps retinal boutons that decrease visual responses with arousal preferentially connect to inhibitory SC neurons, which invert the effects of arousal in their downstream neurons. A similar specificity has been suggested to explain the effects of acetylcholine, which reduces visual responses in SC neurons but typically facilitates transmitter release in retinal afferents (Binns and Salt, 2000). Another explanation is that the effects of arousal in SC neurons are shaped not only by their retinal input but also by neuromodulators acting directly on SC neurons (Binns, 1999).

We do not know what advantages might be obtained by making visual responses vary with behavioral state and introducing this variation as early as in the output of the retina. Moreover, we do not know why reducing both responsiveness and selectivity for orientation and direction would be advantageous during arousal. Theories of efficient coding suggest that the retina is attuned to the statistics of natural scenes and to the behavioral needs (Barlow, 1953, Salisbury and Palmer, 2016, Srinivasan et al., 1982). Both factors change when the animal moves or becomes aroused, and the retina may accommodate this change by adapting its encoding strategy. Our methods to image retinal synapses in the intact brain during behavior may help clarify these open questions.

## STAR★Methods

### Key Resources Table

**Table:** 

REAGENT or RESOURCE	SOURCE	IDENTIFIER
**Bacterial and Virus Strains**		

AAV2/1.Syn.GCaMP6f.WPRE.SV40	University of Pennsylvania Viral Vector Core	AV-1-PV2822
Plasmid: SyGCaMP6f	Laboratory of Leon Lagnado	N/A
AAV-hSyn1-GCaMP6f-P2A-nls-dTomato	Addgene	51085

**Deposited Data**		

Preprocessed data	This paper, (Schröder et al., 2020)	https://rdr.ucl.ac.uk/collections/Arousal_modulates_retinal_output/4934931

**Experimental Models: Organisms/Strains**		

Mouse: C57BL/6J	https://www.jax.org/strain/000664	RRID:IMSR_JAX:000664
Mouse: Gad2-IRES-Cre	https://www.jax.org/strain/010802	RRID:IMSR_JAX:010802
Mouse: Ai9	https://www.jax.org/strain/007909	RRID:IMSR_JAX:007909
Mouse: PV-Cre	https://www.jax.org/strain/008069	RRID:IMSR_JAX:008069
Mouse: Ai32, RCL-ChR2(H134R)/EYFP	https://www.jax.org/strain/012569	RRID:IMSR_JAX:012569
Mouse: Emx1-IRES-Cre	https://www.jax.org/strain/005628	RRID:IMSR_JAX:005628
Mouse: Ai38	https://www.jax.org/strain/014538	RRID:IMSR_JAX:014538

**Software and Algorithms**		

MATLAB	MathWorks	N/A
Suite2p	Pachitariu et al. (2016b)	https://github.com/cortex-lab/Suite2P
ScanImage	Pologruto et al. (2003)	ScanImage 4.2
Kilosort2	Pachitariu et al. (2016a)	N/A
Eye tracking	https://github.com/mkrumin/EyeTracking	https://github.com/mkrumin/EyeTracking
SHARP-Track	Shamash et al. (2018)	https://github.com/cortex-lab/allenCCF
PsychToolBox	PsychToolBox	http://psychtoolbox.org
Study specific analysis code	This paper	https://github.com/sylviaschroeder/schroeder-et-al-2020

### Resource Availability

#### Lead Contact

Further information and requests for resources should be directed to and will be fulfilled by the Lead Contact, Sylvia Schröder (sylvia.schroeder@ucl.ac.uk).

#### Materials Availability

This study did not generate new unique reagents.

#### Data and Code Availability

The pre-processed data generated in this study are available at https://doi.org/10.5522/04/c.4934931 (Schröder et al., 2020); code to analyze pre-processed data is available at https://github.com/sylviaschroeder/schroeder-et-al-2020. The raw data are available on reasonable request.

### Experimental Model and Subject Details

All procedures were conducted in accordance with the UK Animals Scientific Procedures Act (1986) under personal and project licenses released by the Home Office following appropriate ethics review.

We used 32 mice: 11 inbred C57BL/6J (https://www.jax.org/strain/000664; 1 male, 10 female) and 21 transgenic mice (13 female, 8 male) were used in this study. For two-photon imaging, we used 15 mice obtained by crossing Gad2-IRES-Cre (https://www.jax.org/strain/010802) and Ai9 (https://www.jax.org/strain/007909). The heterozygous offspring expressed TdTomato in glutamate decarboxylase 2-positive (GAD2+) cells to identify inhibitory neurons. For optogenetic inactivation of V1, we used 5 mice obtained by crossing PV-Cre (https://www.jax.org/strain/008069) and Ai32 (RCL-ChR2(H134R)/EYFP, https://www.jax.org/strain/012569). The heterozygous offspring expressed ChR2 in parvalbumin-positive cells. For widefield imaging, we used one heterozygous mouse resulting from crossing Emx1-IRES-Cre (https://www.jax.org/strain/005628) and Ai38 (https://www.jax.org/strain/014538). Animals were 6-41 weeks old at the time of surgery with a mean weight of 27.0 g (19.3-51.6 g) and were used for experiments up to the age of 54 weeks. Mice were kept on a 12-h light: 12-h dark cycle. Most animals were single housed after the first surgery.

### Method Details

#### Surgical procedures

Animals were anesthetized with isoflurane (Merial) at 3.5% for induction, and 1%–2% during surgery. Carprofen (5 mg/kg; Rimadyl, Pfizer) was administered subcutaneously for systemic analgesia, and dexamethasone (0.5 mg/kg; Colvasone, Norbrook) was administered as an anti-inflammatory agent to prevent brain swelling. The scalp was shaved and disinfected, and local analgesia (Lidocaine, 6 mg/kg, Hameln pharmaceuticals ltd) was injected subcutaneously under the scalp prior to the incision. Eyes were covered with eye-protective gel (Chloramphenicol, Martindale Pharmaceuticals Ltd). After the animal was placed into a stereotaxic apparatus (5% Lidocaine ointment, TEVA UK, was applied to ear bars), the skin covering and surrounding the area of interest was removed, and the skull was cleaned of connective tissue. A custom made headplate was positioned above the area of interest and attached to the bone with Superbond C&B (Sun Medical). Throughout all surgical procedures, the animal was kept on a heating pad to stabilize body temperature at 37°C. Subcutaneous injections of 0.01 ml/g/h of Sodium Lactate (Hartmann’s solution) were given. After the surgery, the animal was placed into a heated cage for recovery from anesthesia. Mice were given three days to recover while being treated with Carprofen.

In animals used for two-photon imaging, a circular 4 mm craniotomy (centered at approximately −4.2 mm AP and 0.5 mm ML from Bregma) was made using a biopsy punch (Kai medical) and a fine-tipped diamond drill (Type 250-012 F, Heraeus). To reduce bleeding from the bone and from the dura we used bone wax and gel foam, and we cooled the area by applying cold cortex buffer. As the posterior SC is covered by a large sinus running through the dura, we permanently pushed the sinus anteriorly to gain optical access to the SC. We first made a cut into the dura directly posterior to the transverse sinus spanning the whole width of the craniotomy. Then we inserted a custom-made implant into the cut and pushed it anteriorly and a few 100 microns down to apply some pressure on the brain and thus stabilize the imaging. The implant was made of a short tube (2.29 mm inner diameter, 1 mm length) made of stainless steel (MicroGroup, Medway, Massachusetts). A 3 mm glass coverslip (#1 thickness, World Precision Instruments) was glued onto the tube to seal the end that was inserted into the craniotomy. A stainless-steel washer was glued onto the other end of the tube. The washer had an inner diameter that fit exactly around the tube and an outer diameter of 5 mm (Boker’s, Minneapolis, Minnesota). All three pieces were glued to each other using a UV curing adhesive (NOA61, Thorlabs). The glass coverslip was slightly larger than the outer diameter of the tube so that it could be slipped underneath the dura. The implant was placed into the craniotomy so that the washer was sitting on top of the skull and provided stability for the implant. The implant was fixed to the skull with Vetbond (3M) and Superbond C&B (Sun Medical). To prevent any dirt from staining the glass coverslip, we filled the tube of the implant with Kwik-Cast (World Precision Instruments), which could be easily removed before imaging.

For two-photon calcium imaging of activity in SC neurons, we injected the virus AAV2/1.Syn.GCaMP6f.WPRE.SV40 (Chen et al., 2013) at a final concentration of 2.30-4.39e12 GC/ml after making the cut into dura. 115-230 nL of the virus was injected 300-500 μm below the brain surface at the posterior edge of the right SC, which abuts the inferior colliculus. The virus was injected at a rate of 2.3 nL every 6 s (Nanoject II, Drummond). The injection pipette was kept in place for about 10 min after the end of the injection. Neurons in this region of the SC represent the lateral and upper periphery of the visual field (Figure S1A,B).

To image retinal boutons in SC, we engineered a virus to express a calcium indicator localized to synaptic boutons, AAV2-SyGCaMP6f, and we injected the virus into the left eye. We cloned a variant of GCaMP6f fused with a localization signal targeting synaptic terminals (SyGCaMP6f) and packaged it into an AAV2/2 vector (Addgene, 51085). This virus restricted GCaMP expression to boutons (Dreosti et al., 2009). To deliver the virus, the animal was anesthetized with isoflurane (Merial) at 3.5% for induction and 1%–2% during the procedure. Carprofen (5 mg/kg; Rimadyl, Pfizer) was administered subcutaneously for systemic analgesia. Pupils were dilated by applying a drop of Mydriacil (Alcon, Surrey, UK) into the eye. We then applied Viscotears (Alcon) onto the eye and placed a 3-5 mm glass coverslip over the eye to allow for good visual access to the retina. Using a Hamilton syringe (5 μl; needles: 34 gauge, RN NDL, 10 mm length, point 4), we injected approximately 3 μl of AAV2/2.hSyn1.SyGCaMP6f.SV40 with a concentration of 2.44 × 10^12^ viral particles/ml into the vitreous humor.

For electrophysiological recordings, craniotomies were performed above the areas of interest (anterior SC and V1, or optic tract). For large craniotomies (3 mm diameter) we increased stability by inserting a honeycomb disk in the craniotomy. The disk was made of polycarbonate with a 3 mm diameter and 175 μm thickness, and had 19 holes of 500 μm diameter each in a hexagonal pattern (laser cut by Laser Micromachining Ltd, Denbighshire, UK). The disk was fixed to the bone with superglue (Powerflex, Loctite).

#### Two-photon imaging

Two-photon imaging was performed using a standard resonant microscope (B-Scope, ThorLabs Inc.) equipped with a 16x, 0.8 NA water immersion objective (N16XLWD-PF, Nikon) and controlled by ScanImage 4.2 (Pologruto et al., 2003). Excitation light at 970-980 nm was delivered by a femtosecond laser (Chameleon Ultra II, Coherent). Multi-plane imaging was performed using a piezo focusing device (P-725.4CA PIFOC, Physik Instrumente, 400 μm range). Laser power was depth-adjusted and synchronized with piezo position using an electro-optical modulator (M350-80LA, Conoptics Inc.). The imaging objective and the piezo device were light-shielded using a custom-made metal cone, a tube, and black cloth to prevent contamination of the fluorescent signal caused by the monitors’ light. Emission light was collected using two separate channels, one for green fluorescence (525/50 nm emission filter) capturing the calcium transients and one for red fluorescence (607/70 nm emission filter) capturing the expression of TdTomato in inhibitory neurons of Gad-Cre x TdTomato mice.

For imaging neurons in SC, we used 3-4 imaging planes separated by 9-30 μm at depths of 15-100 μm from the surface of SC. The field of view spanned 340-640 μm in both directions at a resolution of 512 × 512 pixels. The frame rate per plane was 6.0-7.5 Hz. One dataset captured a single plane at a resolution of 1024 × 1024 pixels, and with a frame rate of 15 Hz.

For imaging retinal boutons in SC we used 5 or 10 imaging planes (with 3 or 6 fly-back planes) with an inter-plane distance of < 2 μm at depths of 8-46 μm from the surface of SC. The field of view spanned 75-135 μm at a resolution of 256 × 256 pixels imaged at a frame rate of 60 Hz (i.e., the time difference between consecutive planes was 16.7 ms); in some datasets the field of view was 42-10 μm at a resolution of 128 × 128 pixels imaged at a frame rate of 120 Hz (i.e., the time difference between consecutive planes was 8.3 ms). Each plane was thus imaged at a rate of 7.5 Hz. Because the calcium indicator was localized to synaptic boutons, fluorescence was not affected by fibers of passage.

A potential confound when imaging brain activity is increased brain movement during arousal, especially when the animal runs. We thus spaced our imaging planes tightly, < 2 μm apart, so that we could track boutons even if the brain moved perpendicular to the planes. Additionally, we simultaneously imaged tdTomato (expressed in GAD-positive SC neurons), which is independent of activity, and we regressed out this signal from the activity-dependent signal.

#### Intrinsic widefield imaging and retinotopic map

To obtain the retinotopic map in Figure S1B, we performed intrinsic imaging using methods described previously (Pisauro et al., 2013). Presentation of stimuli (periodic moving bars) and data analysis were as described in the paper.

#### Eletrophysiology

Recordings were made using Neuropixels electrode arrays (Jun et al., 2017). Probes were mounted to a custom 3D-printed piece and affixed to a steel rod held by a micromanipulator (uMP-4, Sensapex Inc.). To allow later track localization, prior to insertion probes were coated with a solution of DiI (ThermoFisher Vybrant V22888 or V22885) or DiO (ThermoFisher Vybrant V22886) by holding 2 μL in a droplet on the end of a micropipette and touching the droplet to the probe shank, letting it dry, and repeating until the droplet was gone, after which the probe appeared pink. Probes had a soldered connection to short external reference to ground; the ground connection at the headstage was subsequently connected to an Ag/AgCl wire positioned on the skull. The craniotomies and the wire were covered with saline-based agar. The agar was covered with silicone oil to prevent drying. In some experiments a saline bath was used rather than agar. Probes were advanced through the agar and the dura, then lowered to their final position at ∼10 μm/sec. Electrodes were allowed to settle for ∼10 min before starting recording. Recordings were made in external reference mode with LFP gain = 250 and AP gain = 500. Data were filtered in hardware with a 300 Hz 1-pole high pass filter and digitized at 30 kHz. Recordings were repeated at different locations on each of multiple subsequent days.

During recordings with optogenetic inactivation of V1, one electrode was placed into SC and a second electrode into V1. In 5 of 6 datasets, both electrodes were recorded simultaneously. The SC electrode entered the brain vertically at −3.7 mm AP and ± 0.6 mm ML from Bregma. Receptive fields of recorded SC neurons were thus more anterior than of SC neurons imaged using two-photon imaging. The V1 electrode entered the brain at −3.6 (or −3.5) mm AP and ± 2.25 (or +3.0) mm ML from Bregma. This electrode was tilted backward from vertical by 20° and tilted away from the midline by 45°. 4 of 6 recordings were performed in the right hemisphere.

For recordings from the optic tract, the electrode entered the brain at −1.7 (or −2.0) mm AP and ± 2.62 (or −1.6) mm ML from Bregma. The electrode was tilted backward from vertical by 10° and tilted away from the midline by 90° so it was aligned with coronal plane of the brain. 3 of 6 recordings were performed in the right hemisphere.

#### Optogenetic inactivation

For optogenetics experiments, 473 nm light was generated by a diode laser (LuxX, Photon Lines Ltd.), coupled into an optic fiber, and then focused to a spot ∼1 mm in diameter on the surface of the brain near the probe. Light intensity was modulated in a 40Hz raised cosine wave pattern, with peak light power at the surface of the brain of approximately 0.9-3 mW. The exact light power for each experiment depended on how strongly neural activity simultaneously measured in V1 was affected. The inactivated region of V1 included the retinotopically matched region of simultaneously recorded SC neurons. Optogenetic inactivation lasted for 700 ms per trial including 100 ms before and after the visual stimulus. In one dataset, the inactivation laser was switched on and off simultaneously with the visual stimulus, which was presented for 2 s. Electrophysiological recordings of SC neurons during V1 inactivation were performed at a depth of ≥ 1 mm from brain surface and a distance of approximately 1 mm from the edge of laser spot above V1. As the distance between laser light at brain surface and the most superficial neurons recorded in SC is thus approximately 1.4 mm, laser power has decreased to < 5% when reaching SC and will thus be too weak to affect neurons in SC (Yizhar et al., 2011, https://web.stanford.edu/group/dlab/cgi-bin/graph/chart.php).

#### Experimental setup and visual stimuli

The mouse was head fixed with a headplate holder that did not obstruct the visual field. For two-photon imaging, the mouse was free to run on an air-suspended Styrofoam ball (20 cm in diameter), whose rotation was measured by two optical computer mice (Dombeck et al., 2010). For electrophysiology, the mouse was free to run on a Styrofoam wheel (15 cm wide, 18 cm diameter), whose rotation was measured by a rotary encoder (1,024 pulses per rotation, Kübler, Germany). The mouse was acclimated to head-fixation for at least three days before the first recording session, stepwise increasing fixation time from 10 min on the first day to 1 h on the last day.

The mouse was surrounded by three computer screens (Iiyama ProLite E1980SD placed ∼20 cm from the mouse’s eyes; or Adafruit, LP097QX1 placed ∼11 cm from the mouse’s eyes; 60 Hz refresh rate for both models) at right angles covering approximately 270 × 70 degrees of visual angle. In some experiments, Fresnel lenses (BHPA220-2-6 or BHPA220-2-5, Wuxi Bohai Optics) were mounted in front of the monitors to compensate for reduction in luminance and contrast at steeper viewing angles relative to the monitors. In some of these experiments, lenses were coated with scattering window film (frostbite, The Window Film Company) to prevent specular reflections. To track the eye contralateral to the recording site (except in 5 of 7 recordings in SC during V1 inactivation and 3 of 6 recordings in optic tract), we illuminated the eye with an infrared LED (850 nm, Mightex SLS-0208-A or Mightex SLS-0208-B). Videos of the eye were captured at 30 Hz with a camera (DMK 23U618 or DMK 21BU04.H, The Imaging Source) equipped with a zoom lens (Thorlabs MVL7000) and a filter (long-pass, Thorlabs FEL0750; or band-pass, combining long-pass 092/52x0.75, The Imaging Source, and short-pass FES0900, Thorlabs).

We presented sinusoidal drifting gratings covering all monitors (full-field), at 100% contrast. Gratings had spatial frequency of 0.08 cycles/deg and temporal frequency of 2 Hz. Gratings were presented for 2 s separated by a gray screen for 3-6 s. Only in some electrophysiological recordings in SC, the duration of the gratings was 0.5 s with an inter-trial interval of 0.5-1.0 s. The stimuli were repeated 15 or 20 times. During two-photon imaging, the red channel of the monitors was switched off to reduce light contamination in the red fluorescence channel.

To map receptive fields, we presented checkerboard images with white, black and mean gray squares with an edge length of 10 visual degrees (in one dataset 4 visual degrees). The stimulus updated at a rate of 6 Hz and was presented for 10 min. In each noise image, each square was randomly assigned its luminance value with a 98% probability of being gray and 1% probabilities of being black and white.

Spontaneous activity was recorded either when screens were gray or during darkness. Grey screens were presented at mean luminance of the monitors. The duration was 10 min for SC neurons and 5 or 10 min for retinal boutons. For recordings during darkness, all monitors were switched off and other light sources in the recording rig were eliminated or covered. The recording setup was enclosed either by a frame tightly covered with black curtains or by a closed box, in order to prevent light in the recording room entering the eyes of the animal. The duration of the recordings were 5, 10, or 20 min for retinal boutons and around 40 min for optic tract axons (data used for correlation analyses was 11.7-43 min long; see below for selection of valid time periods).

#### Photoisomerization with varying pupil size

To provide estimates of the range of light intensities expected at the level of photoreceptors with changes of pupil size, we estimated photoisomerization per cone per second (R/cone/s) to range between 2.4-78.3e6 at a wavelength of 500 nm. These estimates are based on published derivations of photoisomerization per second in the mouse eye (Lyubarsky et al., 2004):

$$\frac{\Phi }{\Delta T}=\pi I\times 1500\times \tau \left(\lambda \right)\frac{S_{pupil}}{S_{retina}}a_{c}\left(\lambda \right)$$

We used the following measures: the extent of the monitors spans 1 solid angle (steradian), the irradiance (*I*) of the monitors is ∼83 cd/m^2^, the transmission (*τ(λ)*) of the pre-photoreceptor media is 0.7 (Lyubarsky et al., 2004), the area of the retina (*S*_*retina*_) is 18 mm^2^ (Lyubarsky et al., 2004), the end-on collecting area of the cone (*a*_*c*_*(λ)*) is 1 μm^2^ (Naarendorp et al., 2010), and the size of the mouse pupil (*S*_*pupil*_) varies between 0.1 and 3.2 mm^2^ (Lyubarsky et al., 2004).

#### Perfusion and histology

Mice were perfused with 4% PFA, the brain was extracted and fixed for 24 hours at 4 C in PFA, then transferred to 30% sucrose in PBS at 4 C. The brain was mounted on a microtome in dry ice and sectioned at 60 μm slice thickness. Sections were washed in PBS, mounted on glass adhesion slides, and stained with DAPI (Vector Laboratories, H-1500). Images were taken at 4x magnification for each section using a Zeiss AxioScan, in three colors: blue for DAPI, green for DiO, and red for DiI.

### Data analysis

#### Preprocessing of two-photon imaging data

All raw two-photon imaging movies were analyzed using Suite2p (implemented in MATLAB, Mathworks) to align frames and detect regions of interest (Pachitariu et al., 2016b). We used the red channel representing TdTomato expressed in all inhibitory neurons to align frames, which yielded better results than alignment using calcium dependent fluorescence. For 22 of 28 datasets, alignment was non-rigid. In the remaining ones rigid alignment was sufficient.

For imaging data of retinal boutons, we also aligned frames in depth, i.e., when the brain moved perpendicular to the imaging planes, fluorescence data from neighboring imaging planes was used to correct this movement. This was possible because imaging planes were very close to each other (< 2 μm) so that fluorescence from any neural structure was detected in multiple planes. First, the target images of all planes (except fly-back planes) were aligned to each other in x and y. Then each frame of the red channel data was aligned to each target image and the similarity to each target image was determined. For each imaging cycle through all planes, the optimal shift of this stack of frames was determined by maximizing the mean similarity between frames and target images. For each imaging stack, a moving average across neighboring planes (width 2-4 planes depending on similarity between planes) was applied resulting in higher similarity across planes. Boutons were analyzed only if they appeared in every cycle, i.e., they never moved outside the imaged volume. The algorithm of alignment throughout depth is implemented in Suite2p. Regions of interest (ROIs) were detected using the aligned frames of the green channel, and were then manually curated using the Suite2p GUI.

Every aligned movie was inspected manually to check for failures in automatic alignment. Failures were corrected using different parameter settings where possible. Misaligned movie frames were discarded (1.1 ± 0.6% of frames per dataset were discarded) and ROIs in unstable regions of the field of view were not considered for further analysis.

Using the aligned movies and detected ROIs resulting from Suite2p analysis, we extracted the fluorescence from the green and the red channel within each ROI. To correct the calcium traces for contamination by surrounding neuropil, we also extracted the fluorescence of the surrounding neuropil for each ROI using the green channel. The neuropil mask resembled a band surrounding the ROI with its inner edge having a distance of 3 microns away from the edge of ROI (for neurons; 1 micron for boutons) and its outer edge having a distance of 30 microns from the edge of the ROI. Pixels belonging to other ROIs were excluded. To correct for contamination, the resulting neuropil trace, N, was subtracted from the calcium trace, F, using a correction factor α: F_c_(t) = F(t) - α∙N(t). The correction factor was determined for each ROI as follows. First, F and N were low-pass filtered using the 8^th^ percentile in a moving window of 180 s, resulting in F_0_ an N_0_. The resulting traces F_f_(t) = F(t)-F_0_(t) and N_f_(t) = N(t)-N_0_(t) were then used to estimate α as described previously (Dipoppa et al., 2018). In short, N_f_ was linearly fitted to F_f_ using only time points when values of F_f_ were relatively low and thus unlikely to reflect neural spiking. F_c_ was then low-pass filtered as above (8^th^ percentile in a moving window of 180 s) to determine F_c,0_. These traces corrected for neuropil contamination were then used to determine ΔF/F = (F_c_(t) - F_c,0_(t)) / max(1, mean_t_(F _c,0_(t)).

To correct for potential brain movements, we used the red fluorescence traces of each ROI to regress out changes in fluorescence that were not due to neural activity. First, we low-pass filtered the red trace of each ROI (8^th^ percentile in a moving window of 180 s) and subtracted it from the unfiltered trace to remove slow drifts and bleaching effects. Second, we applied a median filter to the resulting red trace (moving median in window of 10 s). Third, this trace was regressed out of ΔF/F.

We avoided sampling the same neurons/boutons (“units” from here on) more than once. First, we disregarded any datasets with field of views overlapping with those of considered datasets. Second, We detected ROI pairs that were close to each other in neighboring imaging planes and that had highly correlated calcium traces (ρ > 0.4 for neurons and ρ > 0.5 for boutons, correlation between traces filtered using a moving median in a window of 5 samples). Only the ROI of each pair with the highest signal-to-noise ratio was used for further analyses. ROIs that had very long-lasting calcium transients (> 25 s) were removed.

#### Spike sorting

Extracellular voltage traces were preprocessed using common-average referencing: subtracting each channel’s median to remove baseline offsets, then subtracting the median across all channels at each time point to remove artifacts. Electrophysiological data collected in SC was spike sorted using Kilosort with standard parameters (Pachitariu et al., 2016a). Data collected in the optic tract was spike sorted using a modification of Kilosort, termed Kilosort2 (available at https://www.github.com/MouseLand/Kilosort2). Kilosort 2 is able to track spikes of a neuron when its location relative to the probe changes, i.e., during drift. In addition, Kilosort2 performs automated splits and merges similar to what a human curator would do based on spike waveform similarity, on the bimodality of the distribution of waveform features, and on the spike auto- and cross-correlograms. After sorting, all automatically-detected spike clusters were curated manually using Phy (https://github.com/kwikteam/phy).

#### Criteria for selection of retinal axons in optic tract

The first step in identifying spiking units that correspond to axons in the optic tract was the histological analysis of the brains. Using the traces of DiI or DiO left behind by the probe, we determined whether the probe passed through the optic tract, and if so, which part and thus which recording sites were located in the optic tract. Using DAPI staining, the optic tract is readily identifiable so that recordings that did not pass the optic tract could easily be discarded. If the probe did pass through the optic tract, we next used SHARP-Track (Shamash et al., 2018) to align each brain slice to a plane through the Allen Mouse Common Coordinate Framework (http://atlas.brain-map.org/) and record the 3D coordinates of manually selected points along the fluorescence track. A line was fitted through the coordinates, resulting in a vector of brain regions the electrode passed through. Next we localized the position of the probe along this vector of brain areas including the matching scaling factor for this vector. In most cases, it was not possible to locate the tip of the probe in the brain slices with precision high enough to align probe position with the vector of brain areas; instead we used physiological features varying along the probe, such as spike rate and spike amplitude, to match those to the identified brain areas along the reconstructed track (Steinmetz et al., 2019). For example, the lower edge of cortical cell layers and the different cell and dendritic layers of hippocampus were often readily identifiable from the recordings. After this alignment, we selected those recording sites that were estimated to pass through the optic tract (36 of 50 recordings).

The second step in identifying optic tract units was the screening for visual responses indicative of retinal axons. The probe was inserted into the brain so that brain areas recorded on sites close to the optic tract were not part of the early visual system, e.g., internal capsule and globus pallidus above, and medial amygdala below optic tracts. These areas were not expected to have strong visual responses with short latencies. To test visual response properties, we presented three kinds of visual stimuli: (1) luminance reversing (flickering) screens with reversal rates of up to 15 Hz to measure visual responsiveness to fast changes in luminance, (2) visual noise consisting of white and black squares on gray background to measure receptive fields, and (3) full-field drifting sinusoidal gratings to measure direction tuning. Only units that showed a clear visual response and temporal modulation to the fastest flickering screens (15 or 7.5 Hz) were considered to be retinal axons. For very few units, no responses to flickering monitors were collected or the firing rate in response to these stimuli were extremely low. In these cases, units were included only if they had well-defined receptive fields with shapes and sizes expected of retinal ganglion cells, short response latencies to grating stimuli (< 50 ms), and unusual spike waveforms (for example like those in Figure 3B2). Of the 1,280 units that were located in the estimated position of the optic tract, 49 units passed our criteria for retinal axons.

The final step of selecting optic tract units was to discard those units that did not pass our criteria for stable and reliable recordings. Because retinal axons are very fine structures electrophysiological recordings from them are prone to artifactual changes in firing rates resulting from electrode drift. The use of densely spaced recording sites on Neuropixels probes (nearest site at most 20 μm apart (Jun et al., 2017)) together with a spike sorting algorithm that automatically tracks recorded units across electrode drift (see above) helped us to account for the consequences of electrode drift. Still, we employed additional criteria to control that no artifacts contaminated the data used to study the correlation between firing rates and running speed. To avoid biases, we did not consider the recorded running speed when applying these criteria. First, low-amplitude clusters were excluded: if the lowest amplitude spikes in the cluster touched the detection threshold at any point, the entire cluster was not analyzed (18 of 49 units were excluded by this criterion), or the affected time periods of the unit were discarded. Second, any units with a significant correlation between firing rate and spike amplitude were excluded as potentially contaminated by electrode drift (5 of 49 units excluded; significance of correlation was determined using the shift test as described below in Correlation analyses).

If after automatic spike sorting the distribution of spike amplitudes was bimodal, we identified and discarded the smaller (contaminating) spikes using an automatic method. To do so, spikes were binned in bins of 100 spikes. For each time point, we determined the most frequent spike amplitude, termed mass amplitude. The vector of mass amplitudes was smoothed (moving average on 3 bins) and all spikes with amplitudes above the current mass amplitude were used to estimate the SD of the distribution of spike amplitudes (for each spike, the mass amplitude at spike time was subtracted; the resulting distribution of positive amplitudes was fit with a half-Gaussian). Any spikes with amplitudes smaller than the respective mass amplitude minus 5-20 SDs (varying between units) were discarded from further analysis. This was done in 10 of 26 units (for 7 units during darkness). Finally, one of these 10 units had to be excluded because the mouse hardly ran during the remaining periods in darkness (< 5%). We thus included 25 units in our correlation analysis.

We then considered the recorded running speeds of the animal and plotted for each unit its mean spike waveform during a running episode and a temporally nearby stationary episode. We checked that spike waveforms did not change between the two episodes, which would point to electrode movement (see Figure 3B for mean spike waveforms during running and stationarity pooled across all spikes of 2 example units recorded during darkness). No further units had to be excluded based on this analysis.

#### Tracking of pupil

We focused on pupil size as measure of arousal because it measures arousal even when the animal did not run. Movie processing was performed offline using custom code written in MATLAB (Mathworks) on a frame-by-frame basis. Briefly, each frame was mildly spatially low-pass filtered to reduce noise and then the pupil contour was detected by a level-crossing edge detector. Then, concave segments (caused by the reflections of the infrared LED or by whiskers) of the estimated pupil contour were discarded, and the position and the area of the pupil were calculated from the ellipse fit to the concave segments of the pupil contour. Eye-blinks were detected by a two-dimensional classifier, based on overall frame intensity (frames with blinks tend to be brighter) and correlation to the average frame (frames with blinks tend to have lower correlation to the average frame). The output of the algorithm was visually inspected, and adjustments to the parameters (e.g., spatial filter strength, level-crossing threshold, boundaries of the blink detection classifier) were made if necessary. The trace of pupil size was then smoothed by using the median in a moving window of 5 samples. Frames with detected blinks were excluded from the analysis.

#### Correlation analyses

For two-photon imaging experiments, traces of running speed and pupil size were interpolated to match the sampling rate of the calcium traces. All signals were then convolved with a Gaussian window with a sigma of 1 s. Correlation strength was determined using Pearson’s correlation. Because both neural activity and pupil/running are temporally autocorrelated, significance was tested by time shifting. Note that for display purposes, the raw traces of two example boutons in Figures 2A and 2B and the two example neurons in Figure 4E were only smoothed with a moving average over 5 samples (0.67 s).

For electrophysiological recordings from retinal axons in the optic tract during darkness, spikes were binned into bins of 133 ms (to match the commonly used sampling rate of 7.5 Hz for calcium imaging), and running traces were interpolated to samples at the same time points. Running traces and firing rates were then convolved with Gaussian window with sigma of 1 s, after slow drifts were subtracted (drift was estimated by 8^th^ percentile in moving window of 180 s). Then the cross-correlogram of the z-scored signals were calculated. If the signals were composed of separate parts of the data (i.e., when certain parts were excluded by our quality criteria measures), cross-correlograms were calculated separately on each part and then averaged. Significance was tested as above by a shift test. If the signals were composed of separate parts, these were concatenated before the shifts.

#### Estimation of responses to gratings

From calcium traces recorded in an SC neuron or a retinal bouton, we quantified response magnitudes to drifting gratings by iteratively fitting a temporal response kernel that is 15 s long and a scaling factor for each presentation of a grating. First, we used a General Linear Model (not to be confused with Generalized Linear Model) to fit one temporal kernel to all trials using the currently estimated scaling factors (initially set to 1). Second, we used the currently estimated temporal kernel and fitted a scaling factor for each trial, again using a General Linear Model. Both steps were repeated until the values of the estimated scaling factors stopped changing across iterations. We used the scaling factors as response strength in each trial.

The reason for using this approach rather than stimulus-triggered averages of the calcium trace was the dynamics of the calcium decay, which in some units was so slow that it extended into the response to the subsequent stimulus. Our kernel fits correct for these calcium residues in stimulus responses and represent the neural responses well (Figure 1H; adjusted R^2^: 0.3 ± 0.004 for boutons, 0.4 ± 0.006 for SC neurons, mean ± SEM). We only included units that showed significant responses to the gratings. To test significance we randomly shifted the response of each unit against the stimulus presentation times. A unit was determined to be *responsive* if the mean square error (MSE) of the fits to the measured responses was smaller than the 95% confidence interval of MSEs resulting from fits to the randomly shifted responses, which was the case for 2,258 of 3,677 detected retinal boutons and 2,015 of 3,753 detected SC neurons.

To estimate response amplitudes from electrophysiological recordings, we calculated firing rates from spikes between stimulus onset and offset.

#### Fitting tuning curves

Two different tuning curves were fitted for responses during small and large pupil trials. To categorize each trial, the trace of pupil size was interpolated to match the sampling times of the neural data (spikes were binned into 5 ms bins) and then thresholded at the median pupil size measured during the experiment. If pupil size was below that threshold for longer than half of the trial, the trial was categorized as small pupil trial, and otherwise as large pupil trial.

Before fitting, we inverted the responses of all units suppressed by gratings. Note that for these units their preferred direction elicited the largest drop in activity.

Responses during the same pupil size were fit to two wrapped Gaussians with peaks separated by 180 degrees, plus an additive offset. Each curve had five parameters: preferred direction, response at preferred direction (on top of offset), tuning width (sigma of Gaussian), direction selectivity ((P-N)/(P+N), where P is response to preferred direction, N is response to null direction), and offset. Tuning width (sigma) was limited to a minimum of the sampling distance between tested grating directions. Fits used the least-squares method.

Tuning curves for small and large pupil were constrained to have equal preferred direction, tuning width, and direction selectivity. First, one tuning curve was fit to all responses independent of pupil size to find good initial parameters for the following fit. Then two tuning curves were fitted for the two pupil sizes under the condition that the three above parameters are the same for both curves.

Mindful of possible interactions between visual stimuli and arousal (Socha et al., 2018), we checked that the proportion of large pupil trials was independent of visual stimulus (boutons: Figure S2C, p = 0.82, ANOVA; SC neurons: Figure S4K, p = 1.00, ANOVA).

During experiments involving V1 inactivation, we fitted two tuning curves during control conditions and two tuning curves during V1 inactivation. The only parameter that was fixed across all four curves was preferred direction, which was determined by first fitting all responses to a single tuning curve. The fitting procedure for data during small and large pupil was the same as described above.

To decide whether the unit was tuned to direction or not, we compared the fitted wrapped Gaussian to a constant fitted across stimuli. The two models were compared using cross-validated explained variance, i.e., the data of one repetition for each stimulus was left out for fitting and predicted responses were then compared to the left-out data (this was repeated for as many times each stimulus was presented). Units were considered tuned if the cross-validated fit with the Gaussian resulted in larger explained variance than the constant fit (1,750 of 2,258 boutons and 1,308 of 2,015 SC neurons).

Responses to preferred direction in untuned units were defined as the constant number fitted to responses to all stimuli. Tuning depth (difference between the maximum and minimum fitted response) was only defined for tuned units.

We quantified differences in tuning between trials where the pupil was small versus trials where the pupil was large using response modulation and tuning depth modulation. Both measures are defined as the difference between large and small pupil relative to the mean: (L-S)/[1/2 (L+S)], where L and S are either the response amplitudes at preferred direction during large and small pupil or the tuning depth during large and small pupil.

#### Direction and orientation selectivity index

The direction and orientation selectivity index of a unit was determined from its mean response amplitudes, *R*_*k*_, to the drifting gratings. Mean amplitudes of units suppressed by gratings were inverted; remaining negative amplitudes were set to zero. Direction selectivity was assessed by scaling unit vectors pointing into the stimulus direction, *α*_*k*_, by the mean amplitudes, then summing these vectors: $R=\sum_{k}R_{k}\cdot e^{i\alpha_{k}}/\sum_{k}R_{k}$. The angle and length of *R* are preferred direction and direction selectivity index (DSI). To determine preferred orientation and orientation selectivity, *α*_*k*_ was first doubled. Preferred directions and orientations determined in this way were used in Figures S2E, S2F, S4P, and S4Q to compare difference during small and large pupil conditions.

#### Mapping of receptive fields

We presented sparse sequences of white and black squares to characterize the receptive fields, using the responses to white squares for the ON subfield and to black squares for the OFF subfield. We used a linear regression model to simultaneously fit two spatio-temporal filters—one for the ON field and one for the OFF field—and a temporal filter to model the effects of running speed on unit’s response. We modeled the influence of running to account for non-stimulus related fluctuations in the neural responses without idiosyncratic choices of high-pass filters of those responses. For the ON field, only the appearance of white squares was considered, for the OFF field only black squares. The fits were regularized by imposing spatial smoothness on the two receptive fields (Smyth et al., 2003), and similarly imposing temporal smoothness on the running filter. We used two different regularization parameters, one for both receptive fields and one for the running filter. The optimal parameters were determined for each unit individually by maximizing the ten-fold cross-validated explained variance for the complete model. Using these parameters, the receptive fields and running filter were then modeled using the complete response trace of each unit. A retinal bouton or SC neuron was considered to have a genuine receptive field if (1) the cross-validated explained variance by its receptive fields (not considering the running filter) was > 0.01 and significant (p < 0.05, shift test), and (2) the optimal regularization parameter was small enough to result in spatially confined receptive fields. Before the fit, all traces (neural and running speed) were resampled or binned to match the presentation times of the stimulus frames, which were updated every 167 ms. To model calcium traces, the receptive fields mapped the optimal stimulus one to three frames before the calcium response. For firing rates from the optic tract, receptive fields mapped the optimal stimulus at the time of spikes and one frame before. For all calcium traces, only contralateral stimuli were considered to minimize the number of modeled parameters. For firing rates, the complete stimulus was considered if the number of pixels was smaller than the number of stimulus frames. The fitted running filter spanned −5 to 5 s relative to the modeled response. Figures 1F and S4A show spatial ON and OFF receptive fields at the time of their maximum. Black outlines show a fit by a two-dimensional Gaussian at half height to the sum of the ON and OFF fields. ON/OFF index was defined as (r_ON_ - r_OFF_)/(r_ON_ + r_OFF_), where r_ON_ and r_OFF_ are the peak values at the ON and OFF fields, where peak is the pixel at the maximum of the mean across both fields. In some boutons one or both of the fields were negative (the bouton was suppressed by white or black squares); for these boutons, we inverted the sign of the responses r before computing the index. If the ON and OFF fields had different signs, the weaker peak value was set to zero.

### Quantification and Statistical Analysis

All statistical tests can be found in Results or figure captions and include distinct samples, i.e., measures at different time points or measures from different units, and no sample was considered multiple times. Reported n values refer to the number of retinal boutons, retinal axons, SC neurons, or V1 neurons there were used in the test. All tests were two-sided, unless otherwise stated.

#### Fisher’s combined probability test

To verify whether the number of cells correlating with a variable exceeded that expected from independent chance, we used Fisher’s method of combined probabilities. If *p*_*c*_ represents the p value for a test of cell *c*, Fisher’s method combines these into a chi-square statistic ${\text{χ}}_{2n}^{2}=-2\sum_{c=1}^{n}\ln\left(p_{c}\right)$, which follows a $\chi_{2n}^{2}$ distribution under the assumption that all p values are independent.

#### Permutation test

Permutation tests were performed by generating surrogate datasets from the measured data, computing the test statistic for each surrogate dataset, and comparing the resulting null distribution to the test statistic of the measured data, i.e., whether or not the measured test statistic falls inside the 2.5-97.5 percentiles of the null distribution. The surrogate datasets were generated by randomly permuting the relevant feature/label across sample points. To test significance of direction and orientation selectivity (DSI and OSI), we permuted the stimulus direction across trials (1,000 times), and then used a one-sided test and a significance level of p = 0.05. To test separation between DSI and OSI (Figure 1K), we permuted OSIs across boutons (10,000 times); to test significance of response modulation and tuning depth modulation by arousal, we permuted the low and high arousal grouping across trials (200 times) and refitted the tuning curves; to test the difference between response modulation during control conditions and V1 inactivation (Figure 4K), we permuted control and inactivation labels across trials (200 times), refitted tuning curves and determined the difference between response modulations between the two conditions.

#### Shift test

Like permutation tests, shift tests were performed by generating surrogate datasets from the measured data. The surrogate datasets were generated by circularly shifting the running or pupil size trace by a random amount of time (500 times). Then Pearson’s correlation with the calcium traces constitute the null distribution. The correlation of the measured data was significant if it fell outside the 2.5-97.5 percentile interval of the null distribution.

#### Circular paired t test

To test whether preferred directions and orientations change between low and high arousal, we first determined the differences between preferences in both conditions (for circular data), and then tested whether the resulting distribution has a mean different from zero, using function circ_mtest of the CircStat toolbox (Berens, 2009).

#### Linear mixed-effects model

To account for dependence between samples (i.e., data from SC neurons or retinal boutons) recorded in the same experimental session or originating from the same animal, we tested linear relationship between variables using linear mixed-effects models (function fitlme in MATLAB, MathWorks). For each test, we started by using session and animal as random-effects on intercept, variables, and the interaction between intercept and variables. We then eliminated non-significant random-effects (e.g., interactions, or animal as random effect), until only significant effects were included.

## Supplemental Information

Document S1. Figures S1–S4

Document S2. Article plus Supplemental Information
