# Supplementary material for: Arousal Modulates Retinal Output
Source: Neuron. 2020 Aug 5;107(3):487–495.e9. doi: 10.1016/j.neuron.2020.04.026 (PMC7427318; doi:10.1016/j.neuron.2020.04.026)
Supplement: Document S1. Figures S1–S4 [file mmc1.pdf]

Neuron, Volume ***107***

Supplemental Information

Arousal Modulates Retinal Output

Sylvia Schröder, Nicholas A. Steinmetz, Michael Krumin, Marius Pachitariu, Matteo Rizzi, Leon Lagnado, Kenneth D. Harris, and Matteo Carandini

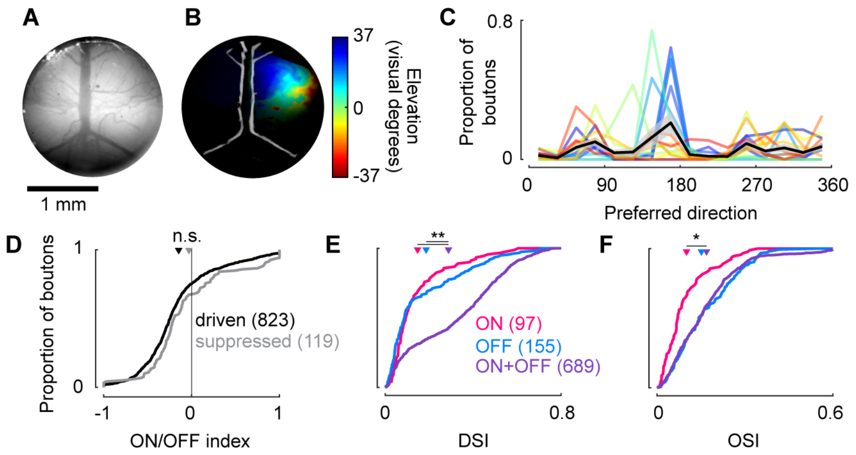

Figure S1. Related to Figure 1. Visual responses of retinal boutons in superior colliculus.

A, Same as in Figure 1D2: view through implant showing SC and inferior colliculus.

B, Retinotopic map (via intrinsic optical imaging) of visual elevation in right SC (same brain as in A). Brightness represents signal-to-noise ratio.

C, Distribution of preferred directions for each recording site (colored) and averaged across recording sites (mean±SEM, black and gray shade).

D, Distribution of ON/OFF indices for boutons driven and suppressed by gratings. Mean ON/OFF indices were not significantly different ($p = 0.1943$, linear mixed-effects model).

E, Distribution of DSIs for “ON”, “OFF”, and “ON+OFF” boutons. DSIs in “ON+OFF” boutons was higher than in “ON” boutons ($p = 1.83e-5$) and higher than in “OFF” boutons ($p = 0.001$).

F, Same, for distribution of OSIs. Orientation selectivity in “ON+OFF” boutons was higher than in “ON” boutons ($p = 0.04$, linear mixed-effects model) but not higher than in “OFF” boutons ($p = 0.30$).

Stars indicate significance (* if $p < 0.05$; ** if $p < 0.01$).

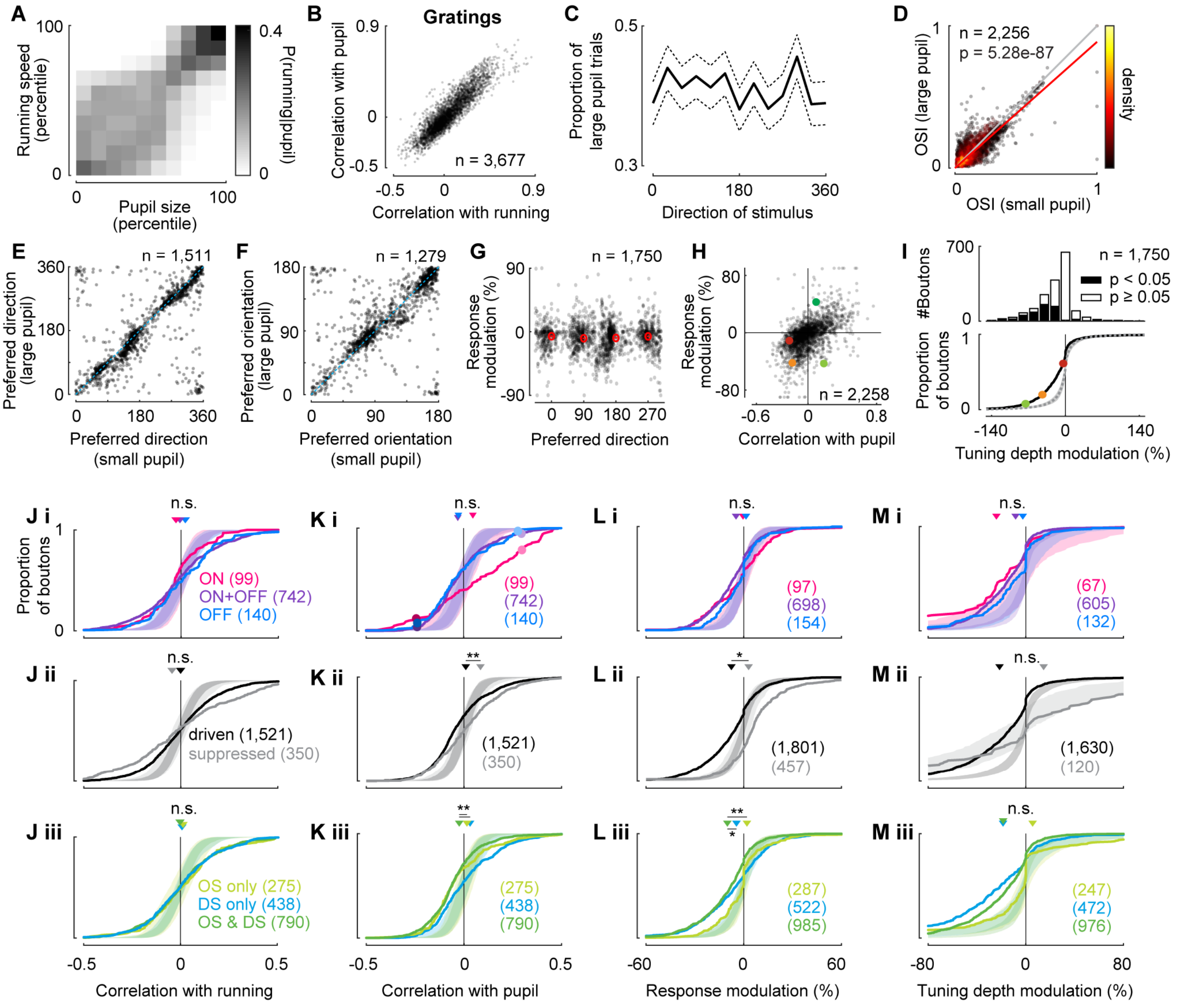

Figure S2. Related to Figure 2. Visual activity in retinal boutons varies with arousal.

A, Distribution of running speed (quantified in percentiles of all measured running speeds) given the simultaneously measured pupil size (quantified in percentiles). Each column sums to 1. The plot shows that running speed and pupil size strongly depend on each other.

B, Correlations of retinal boutons with pupil size versus correlations with running speed during presentation of gratings. Pearson's $\rho = 0.91$ ($p < 1e\text{-}20$).

C, Proportion of large pupil trials (mean±SEM) for each direction of movement of grating.

D, Orientation selectivity index (OSI) during small versus large pupil for each bouton. Red line shows linear regression fit (linear mixed-effects model without intercept).

E, Preferred directions during small versus large pupil. Only boutons with significant DSI during small and large pupil were included.

F, As in E but for preferred orientations of orientation selective boutons.

G, Preferred directions versus response modulations. Boutons were divided into four groups depending on their preferred directions. Red circles and bars mark response modulations (mean±SEM) of each group.

H, Correlations with pupil size during presentation of gratings (visually driven) versus response modulations. Dots show values of example boutons in Figure 2G.

I, Distribution of tuning depth modulations for tuned boutons only. Dots (bottom) mark values of *boutons 1, 2*, and *4* in Figure 2G.

J, Distribution of correlations with running in darkness for "ON", "OFF", and "ON+OFF" boutons (Ji), for boutons driven vs. suppressed by gratings (Jii), and for boutons selective for orientation only (OS),

direction only (DS), and for both orientation and direction (OS & DS) (Jiii). Numbers of boutons of each type are in brackets. Triangles mark mean values for each type after accounting for effects of sessions and mice (using linear mixed-effects model).

K, As in J, for correlations with pupil size during gratings. Correlations are significantly larger for boutons suppressed vs. driven by gratings ($p = 0.0043$, Kii), for OS vs. OS & DS boutons ($p = 0.0158$, Kiii), and for DS vs. OS & DS boutons ($p = 0.0155$, Kiii).

L, As in J, for response modulations. Response modulations are significantly larger for boutons suppressed vs. driven by gratings ($p = 0.0235$, Lii), for OS vs. OS & DS boutons ($p = 3.34e-5$, Liii), and for DS vs. OS & DS boutons ($p = 0.0305$, Liii).

M, As in J, for tuning depth modulations.

Significant differences between types are marked by stars (* if $p < 0.05$; ** if $p < 0.01$).

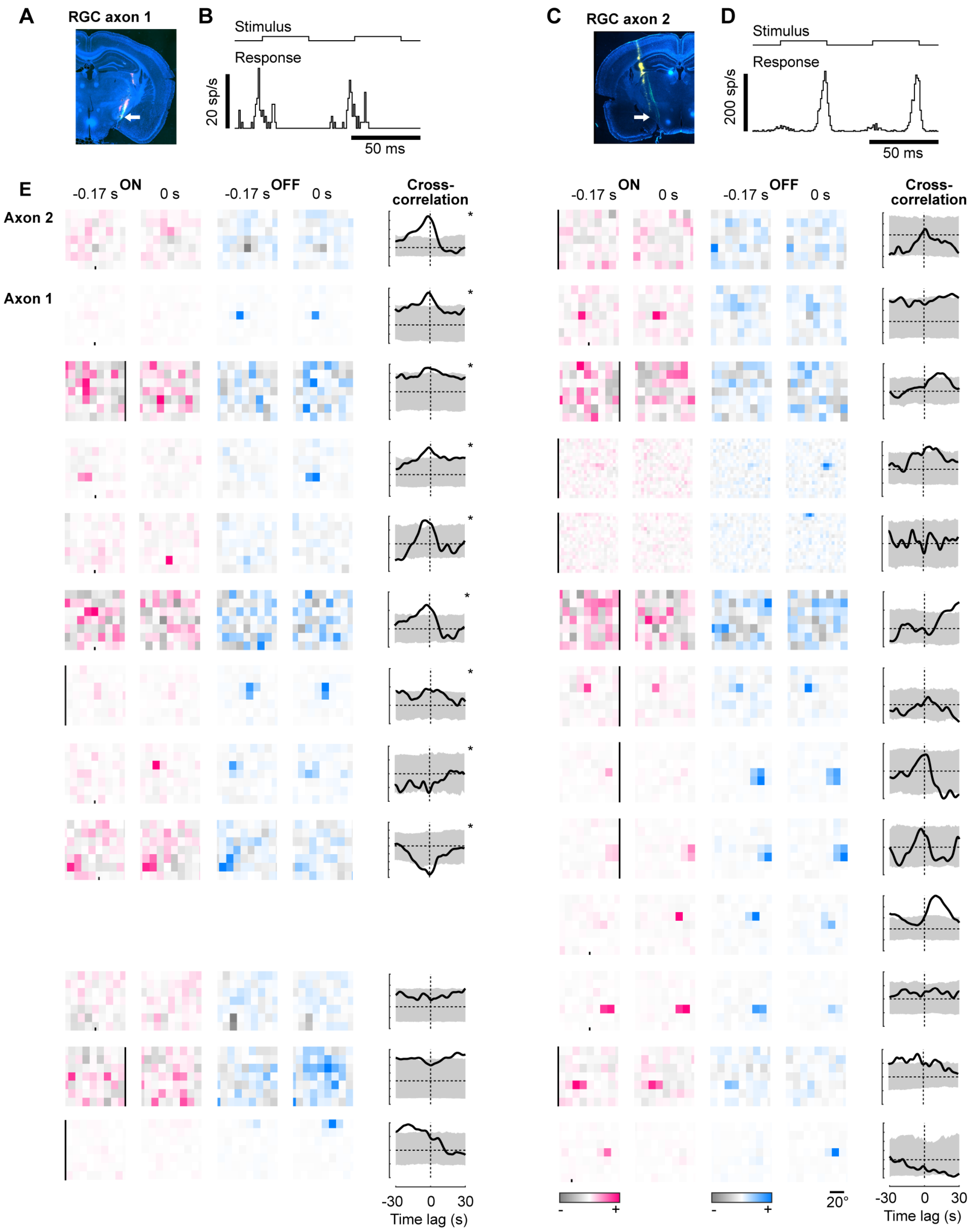

Figure S3. Related to Figure 3. Effect of arousal is present in firing rates of retinal ganglion cells.

A, Coronal brain slice with tracks of multiple recordings (green and red), arrow points to recording of axon 1.

B, Average response (bottom trace) of axon 1 to presentation of full-field reversing (black/white) stimulus (top trace). Luminance changed every 33.3 ms. Note that response is not necessarily to nearest change in luminance but possibly to the previous change.

C,D, Same plots as A,B for axon 2.

E, Receptive fields and cross-correlograms for the 25 recorded units in the optic tract. Images depict ON and OFF fields at time of stimulus presentation (0 s) and one stimulus frame earlier (-0.17 s). Location of receptive field maps is marked in leftmost subfield (ON, -0.17 s) relative to monitor edges at -135° (marked by bar to left of subfield), at +135° (marked by bar to right of subfield), or relative to the center of the visual field (marked by a tick mark). Cross-correlograms also show 2.5-97.5 percentile interval of null distribution (gray), which was used to determine significance at time lag of 0 s

(significant correlations marked by stars). Horizontal dotted lines mark zero correlations. Tick marks on y-axis are separated by 0.1.

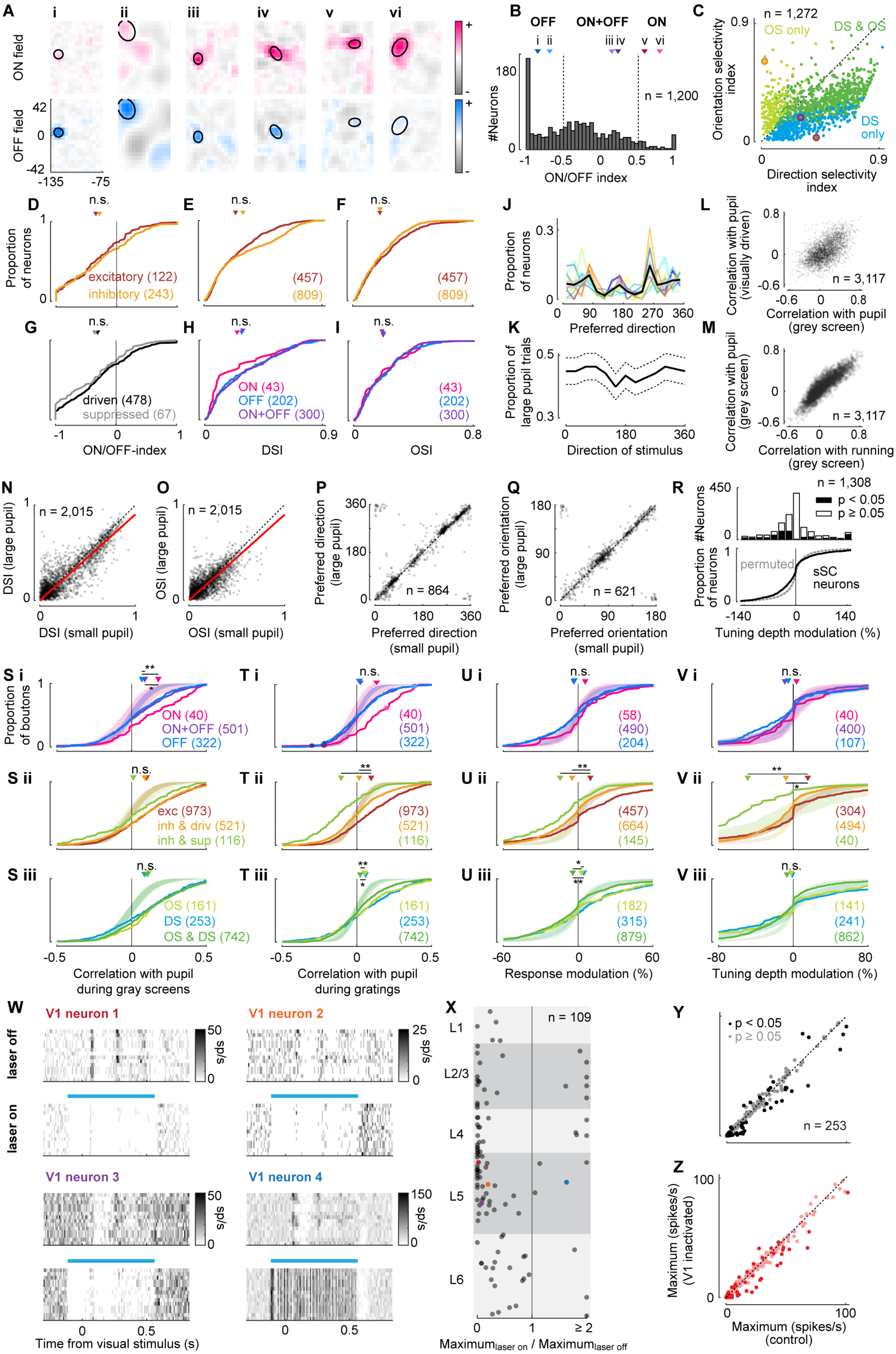

A
i ii iii iv v vi
ON field
OFF field
42
0
-42
-135 -75
B
OFF ON+OFF ON
i ii iii iv v vi
180
#Neurons
0
n = 1,200
-1 -0.5 0 0.5 1
ON/OFF index
C
n = 1,272
OS only
DS & OS
DS only
Orientation selectivity index
0.9
0
0 0.9
Direction selectivity index
D
n.s.
Proportion of neurons
excitatory (122)
inhibitory (243)
E
n.s.
(457)
(809)
F
n.s.
(457)
(809)
G
n.s.
driven (478)
suppressed (67)
-1 0 1
ON/OFF-index
H
n.s.
ON (43)
OFF (202)
ON+OFF (300)
0 0.9
DSI
I
n.s.
(43)
(202)
(300)
0 0.8
OSI
J
Proportion of neurons
0.3
0
0 90 180 270 360
Preferred direction
K
Proportion of large pupil trials
0.5
0.3
0 180 360
Direction of stimulus
L
Correlation with pupil (visually driven)
0.8
0
-0.6
n = 3,117
-0.6 0 0.8
Correlation with pupil (grey screen)
M
Correlation with pupil (grey screen)
0.8
0
-0.6
n = 3,117
-0.6 0 0.8
Correlation with running (grey screen)
N
n = 2,015
DSI (large pupil)
DSI (small pupil)
O
n = 2,015
OSI (large pupil)
OSI (small pupil)
P
Preferred direction (large pupil)
n = 864
Preferred direction (small pupil)
Q
Preferred orientation (large pupil)
n = 621
Preferred orientation (small pupil)
R
n = 1,308
#Neurons
450
p < 0.05
p ≥ 0.05
Proportion of neurons
permuted
sSC neurons
-140 0 140
Tuning depth modulation (%)
S i
Proportion of boutons
**
*
ON (40)
ON+OFF (501)
OFF (322)
T i
n.s.
(40)
(501)
(322)
U i
n.s.
(58)
(490)
(204)
V i
n.s.
(40)
(400)
(107)
S ii
n.s.
exc (973)
inh & driv (521)
inh & sup (116)
T ii
**
(973)
(521)
(116)
U ii
**
(457)
(664)
(145)
V ii
**
*
(304)
(494)
(40)
S iii
n.s.
OS (161)
DS (253)
OS & DS (742)
-0.5 0 0.5
Correlation with pupil during gray screens
T iii
**
*
(161)
(253)
(742)
-0.5 0 0.5
Correlation with pupil during gratings
U iii
*
**
(182)
(315)
(879)
-60 0 60
Response modulation (%)
V iii
n.s.
(141)
(241)
(862)
-80 0 80
Tuning depth modulation (%)
W
V1 neuron 1
V1 neuron 2
laser off
laser on
50
25
sp/s
0
V1 neuron 3
V1 neuron 4
50
150
0 0.5
Time from visual stimulus (s)
X
n = 109
L1
L2/3
L4
L5
L6
0 1 ≥ 2
Maximum laser on / Maximum laser off
Y
p < 0.05
p ≥ 0.05
n = 253
Z
Maximum (spikes/s) (V1 inactivated)
100
0
0 100
Maximum (spikes/s) (control)

Figure S4. Related to Figure 4. Visual responses and effect of arousal on neurons in superior colliculus.

A, ON (top) and OFF (bottom) receptive fields of six neurons (*i-vi*). Ellipses outline receptive fields at half height.

B, Distribution of ON/OFF indices across all neurons (same as in Figure 4B). ON/OFF indices of examples in A are marked by triangles.

C, Orientation and direction selectivity indices of neurons that are only selective to orientation (light green), only selective to direction (blue), or selective to both orientation and direction (dark green). Circles mark values of examples 1-3 in Figure 4F. Selectivity to stimulus direction and orientation was slightly higher in SC neurons compared to retinal boutons ($p = 2.07e-9$ for DS, $p = 1.25e-39$ for OS, Wilcoxon rank sum test). The separation between orientation and direction selectivity in SC neurons has been observed before (de Malmazet et al., 2018), but was much weaker compared to that in retinal boutons (Figure 1K).

D, Distribution of ON/OFF indices for excitatory and inhibitory neurons. Mean indices (resulting from linear mixed-effects model) are marked by triangles. Number of neurons depicted in brackets.

E, Distribution of DSIs for excitatory and inhibitory neurons.

F, Distribution of OSIs for excitatory and inhibitory neurons.

G, Same as in D, for neurons that are driven or suppressed by gratings.

H, Same as in E, for “ON”, “OFF”, and “ON+OFF” neurons.

I, Same as in F, for “ON”, “OFF”, and “ON+OFF” neurons.

J, Distribution of preferred directions for each recording site (colored) and averaged across recording sites (mean±SEM, black and gray shade).

K, Proportion of large pupil trials (mean±SEM) was not significantly different across different directions of movement of gratings ($p = 1.00$, ANOVA).

L, Correlations with pupil size during presentation of gray screens versus gratings. Pearson’s $\rho = 0.58$ ($p = 2.82e-277$).

M, Correlations with running versus pupil size during presentation of gray screens. Pearson’s $\rho = 0.89$ ($p < 1e-20$).

N, DSIs during small versus large pupil for each neuron. Red line shows linear regression fit (linear mixed-effects model without intercept).

O, Same as in N, for OSIs.

P, Preferred direction during small versus large pupil. Only neurons with significant DSI during small and large pupil were included.

Q, Same as in P, for preferred orientation.

R, Distribution of tuning depth modulations for tuned neurons only. Tuning depth decreased in 20% of SC neurons, and increased in 10% of the neurons ($p < 0.05$, permutation test).

S, Distribution of correlations with pupil size during presentation of gray screens for “ON”, “OFF”, and “ON+OFF” neurons (Si), for excitatory neurons and inhibitory neurons that are driven or suppressed by gratings (Sii), and for neurons selective for orientation (OS), directions (DS), and for both orientation and direction (OS & DS) (Siii). Numbers of neurons of each type are in brackets. Triangles mark mean values for each type after accounting for effects of sessions and mice (using linear mixed-effects model). Significant differences between types are marked by stars (* if $p < 0.05$; ** if $p < 0.01$). Correlations are significantly larger for “ON” than “ON+OFF” neurons ($p = 0.0192$, Pi) and “OFF” neurons ($p = 0.0046$), and for “ON+OFF” vs. “OFF” neurons ($p = 0.0018$).

T, As in S, for correlations with pupil size during gratings. Triangles mark mean correlations for each cell type. Dots in Ti mark correlation values of example neurons from A (color code as in B). Like the retinal boutons, arousal decreased responses in some “ON” SC neurons and increased responses in some “OFF” SC neurons speaking against the hypothesis that effects by arousal can be explained by changes in pupil size alone (Ti). Correlations are significantly larger for excitatory than inhibitory neurons that are driven by gratings ($p = 1.09e-21$, Tii) and inhibitory neurons that are suppressed by

gratings ($p = 3.19e-22$, Tii). Also, correlations are significantly larger for OS vs. OS & DS neurons ($p = 0.0074$, Tiii), and for DS vs. OS & DS neurons ($p = 0.0335$, Tiii).
U, As in S, for response modulations. Response modulations are significantly larger for excitatory than inhibitory neurons that are driven by gratings ($p = 1.45e-8$, Uii) and inhibitory neurons that are suppressed by gratings ($p = 1.19e-7$, Uii). Also, response modulations are significantly larger for DS vs. OS ($p = 0.0384$, Uiii) and OS & DS neurons ($p = 0.0049$, Uiii), and for OS vs. OS & DS neurons ($p = 0.0148$, Uiii).
V, As in S, for tuning depth modulations. Tuning depth modulations are significantly larger for excitatory than inhibitory neurons that are driven by gratings ($p = 0.0143$, Vii) and inhibitory neurons that are suppressed by gratings ($p = 1.72e-4$, Vii).
W, Mean firing rates of four layer 5 (L5) V1 neurons during control conditions (top) and V1 inactivation (bottom). Each row shows a single trial response to the same grating, presented for 0.5 s.
During V1 inactivation, the laser was switched on from 0.1 s before to 0.1 s after stimulus presentation (blue line above plot). Note that neuron 4 was strongly activated when the laser was on, indicative of a putative parvalbumin-positive, inhibitory neuron.
X, Ratio of visual response to preferred stimulus during V1 inactivation and control condition, plotted against the depth of the neurons within V1. Depth is scaled between 0 (surface of V1) and 1 (bottom of layer 6). Neurons with ratios larger than 1 are putative inhibitory neurons that are excited by laser stimulation. Layer 5 of V1 contains neurons projecting to SC. Colored dots: example neurons in W.
Y,Z, Maximum amplitudes in response to gratings of all SC neurons during control condition vs. V1 inactivation. Pupil was either small (Y) or large (Z). Darker dots: cells with significant response changes during V1 inactivation ($p < 0.05$, permutation test). Maximum amplitudes during control condition are on average 12% (when pupil small, Y) or 10% (when pupil large, Z) larger than during V1 inactivation (relative to mean amplitude for control and V1 inactivation, $p = 0.0023$ for small pupil, $p = 0.0039$ for large pupil, Wilcoxon signed rank test).
